# Relevance of Oxygen Concentration in Stem Cell Culture for Regenerative Medicine

**DOI:** 10.3390/ijms20051195

**Published:** 2019-03-08

**Authors:** Cristina Mas-Bargues, Jorge Sanz-Ros, Aurora Román-Domínguez, Marta Inglés, Lucia Gimeno-Mallench, Marya El Alami, José Viña-Almunia, Juan Gambini, José Viña, Consuelo Borrás

**Affiliations:** 1 Freshage Research Group- Department of Physiology, Faculty of Medicine, University of Valencia, Avenida Blasco Ibañez 15, CIBERFES, INCLIVA, 46010 Valencia, Spain; cristina.mas@uv.es (C.M.-B.); sanzros@alumni.uv.es (J.S.-R.); aurora.roman@ext.uv.es (A.R.-D.); Lucia.Gimeno@uv.es (L.G.-M.); elalamimarya@gmail.com (M.E.A.); juan.gambini@uv.es (J.G.); jose.vina@uv.es (J.V.); 2 Freshage Research Group- Department of Physiotherapy, Faculty of Physiotherapy, University of Valencia, CIBERFES, INCLIVA, 46010 Valencia, Spain; marta.ingles@uv.es; 3 Master’s Program in Oral Surgery and Implant Dentistry, Faculty of Medicine and Dentistry, University of Valencia, 46010 Valencia, Spain; josevinaalmunia@gmail.com

**Keywords:** aging, redox, physiological oxygen concentration, environmental oxygen concentration, physioxia, senescence

## Abstract

The key hallmark of stem cells is their ability to self-renew while keeping a differentiation potential. Intrinsic and extrinsic cell factors may contribute to a decline in these stem cell properties, and this is of the most importance when culturing them. One of these factors is oxygen concentration, which has been closely linked to the maintenance of stemness. The widely used environmental 21% O_2_ concentration represents a hyperoxic non-physiological condition, which can impair stem cell behaviour by many mechanisms. The goal of this review is to understand these mechanisms underlying the oxygen signalling pathways and their negatively-associated consequences. This may provide a rationale for culturing stem cells under physiological oxygen concentration for stem cell therapy success, in the field of tissue engineering and regenerative medicine.

## 1. Physiological Oxygen Levels In Vivo

Very much importance was given to the balance of nutrients, growth factors and pH buffers used to grow cells in vitro [1]. However, very little attention was given to the oxygen concentration in the culture media as it was assumed that the ambient air (21% O_2_/21 kPa/160 mmHg) was adequate for cell growth [2]. Room air oxygen concentration is still widely used in vitro by the traditional incubators; however, at the tissue level, oxygen concentrations in vivo are significantly lower, limiting its inherent toxicity. According to the extensive review performed by Keeley and Mann, cell culture undertaken under room air conditions falls short of replicating this protection in vivo [3]. Indeed, adult tissues experience a wide range of oxygen tensions that are considerably different from the inhaled ambient oxygen tensions. The partial pressure of oxygen (PO_2_) progressively decreases after it enters in the lungs and is transported by blood to reach the tissue where the final physiological oxygen concentrations are reached. According to air routes in human organisms, the most oxygenated organs will be lungs, stomach and skin as they are in direct contact with air, followed by the own vasculature as it transports the air in blood. Finally, air will reach every organ, where an average of 2–9% O_2_/14–65 mmHg is currently accepted [4,5], and the actual oxygen concentration in situ strongly depends on the vascularization of the tissue and its metabolic activity [6].

Unlike most cell types, lung epithelial cells experience a high PO_2_ physiologically and are separated from gaseous oxygen by a thin layer of air–surface–liquid. As reported in the bibliography, average PO_2_ values for tracheal, bronchial, bronchiolar and alveolar epithelial cells are 13–14% O_2_ [7,8,9,10,11,12], which correlates with the proximity of inspired air. Cutaneous PO_2_ is known to be directly proportional to its own blood flow [13], indirectly proportional to temperature [14], and inversely proportional to epidermal thickness [15]. Furthermore, as stated before, the skin has two oxygen sources, the atmosphere and the microvasculature. In standard conditions, PO_2_ at the subcutaneous level has been reported to range from 3% to 8% O_2_ [16,17]. Below the skin, adipose tissue can be found. PO_2_ values of 7.5% O_2_ have been described in the arm [18] and in the abdomen [19,20,21] of lean patients. Regarding the vascular network, oxygen supply to the vascular wall occurs mainly by diffusion from the lumen (blood PO_2_ is 12% O_2_); thus, the vessel wall PO_2_ decreases between luminal and adventitial layers. Moreover, the thickness of the wall will also interfere in the oxygen supply. Estimating the exact PO_2_ values experienced by all cell types in the vascular wall: endothelial cells, smooth muscle and fibroblasts are pretty hard; however, several studies report a PO_2_ range of 3–10% O_2_ depending on the distance from the lumen [22,23,24,25,26].

Well irrigated parenchymal organs have a wider range of PO_2_, depending on the depth location inside the tissue. The heart is a highly metabolically active tissue with large oxygen requirements. The coronary microvasculature penetrates the myocardium and, as a result, a gradient of PO_2_ has been observed between the superficial epicardium, the deep myocardium and individual myocytes. Such levels range 2–6% O_2_ [27,28,29,30,31,32,33]. Similar to other major and well irrigated organs, the cerebral vasculature extends superficially throughout the brain and penetrates the inner layer of the cortex [34]. Accordingly, different PO_2_ values have been recorded, decreasing proportionally to the depth of the tissue: from 5% O_2_ in the superficial cortex [35,36,37] to 3% O_2_ in the deep white matter [38,39]. Remarkably, studies performed in rats recorded values to drop to 0.5% O_2_ in the deeper regions (hypothalamus, hippocampus and midbrain) [40]. The liver is a relatively well oxygenated organ as blood entering through the hepatic artery is at 12% O_2_ and blood entering through the portal vein is at 6.5% O_2_ [41]. However, blood exiting the hepatic vein reported a PO_2_ value of 5% O_2_ [42]. Indeed, PO_2_ values of 4–7% O_2_ have been reported for liver parenchyma [43,44,45,46]. Kidneys receive a 20% of cardiac output, which is disproportionate compared with other organs with high blood flow. As found in the bibliography, renal cortical PO_2_ ranges 4–9.5% [47] and this value decreases until 2% O_2_ when reaching the medulla [48,49]. Pancreas is also a well irrigated organ, even though 10–15% of the total organ blood flow irrigates the Islets of Langerhans, which constitute 1–2% of total pancreatic tissue. Therefore, the exocrine pancreas has been reported to receive PO_2_ values that range 4.6–2.7% O_2_, whereas endogenous β cells PO_2_ values recorded are higher (5–6% O_2_) [50,51]. Measurement of digestive tract PO_2_ should be divided into three sections: stomach, small and large intestines. In the case of the stomach, no difference has been recorded between the three layers, and average PO_2_ values from 6 to 10% O_2_ have been recorded [52,53]. Then, oxygen levels show a clear decrease along the gastrointestinal tract, reaching its lower levels in the colon. This is in accordance with the presence of anaerobic bacterial flora in the final segments of the gut. Small intestine PO_2_ values are 2–5%, 3–6% and 5–9% O_2_ for lumen, mucosa and serosa layers, respectively [54]. Large intestine PO_2_ values are lower, and have been recorded in a range 0–2% O_2_ for lumen and mucosa layer and 4–6% O_2_ for the serosa layer [55].

The uterus is a highly dynamic organ that experiences changes throughout the menstrual cycle. During the estrogenic phase, intrauterine PO_2_ has been reported to be 2.5% O_2_ in humans [56]. This low PO_2_ rises rapidly within the uterine wall (myometrium/decidua) upon conception or during luteus phase in line with increases in blood flow. Another very dynamic organ is the skeletal muscle. For this reason, muscular PO_2_ should be addressed in resting and in contraction where oxygen requirements may increase two orders of magnitude above rest. A recent study reported that baseline interstitial PO_2_ in the resting spinotrapezius muscle in humans was 16 ± 2 mm Hg (~2.5% O_2_) [57]. During the early contraction, transient interstitial PO_2_ fell quasi–exponentially to values approaching intramyocyte PO_2_ found in humans performing moderate exercise (i.e., approximately 5 mm Hg/0.6% O_2_) [58,59].

**Figure 1:**
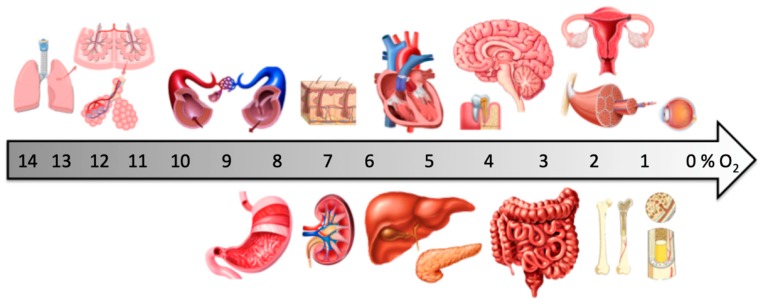
Oxygen partial pressure in tissues.

Less irrigated organs receive less oxygen and thus their PO_2_ is significantly lowered. Bone marrow, as a primary source for mesenchymal and hematopoietic stem cells, can be isolated from sternum and iliac crest, where PO_2_ values have been reported to be 5.4% O_2_ and 7% O_2_, respectively, in humans [60,61,62,63,64,65]. However, values of 10 mmHg (1.5% O_2_) in bone marrow have also been reported [66]. Nevertheless, fully mineralized bone tissue exists at a very low PO_2_, such as 1.4% O_2_ in cartilage [67]. Finally, in the human eye (retina, corpus vitreous), PO_2_ values reported a range from 1% to 5% O_2_ [68,69]. Figure 1 illustrates the average oxygen pressure for each of the aforementioned tissues.

## 2. Stem Cell Niches in Adult Tissues

The concept of a stem cell niche was first proposed by Schofield in 1978 as a physiologically restricted microenvironment that supports stem cells [70]. The stem cell niches can be defined as specific anatomic locations that regulate their participation in tissue generation, maintenance and repair [71]. The stem cell niche is a complex, heterotypic, and dynamic structure, which includes supporting extracellular matrix, neighbouring niche cells, secreted soluble signalling factors (such as growth factors and cytokines), physical parameters (such as shear stress, tissue stiffness, and topography), and environmental signals (metabolites, hypoxia, inflammation, etc.) [72,73]. Stem cells, blood vessels, nerves, matrix glycoproteins and the three-dimensional space forming this unit provide a highly specialized microenvironment. Contact and communication between these elements is critical for stem cell self-renewal and cell fate regulation, thus rendering tissue homeostasis and regeneration.

Several niches have been identified in many adult tissues:

- In lungs, two main stem cell populations have been described. Basal stem cells (BSCs) have the capacity to self-renew and to form secretory and ciliated cells [74,75,76]. Distal alveolar stem cells (DASCs), which are present in the distal airways after H1N1 influenza virus infection and have the capacity to replace injured alveolar cells [77,78].
- In the skin, epithelial stem cells are found in the bulge area of the hair follicles [79], while the exact components of skin niche have not been fully identified yet, although critical regulatory cues derive from the dermal papilla. These stem cells are important in regeneration of hair follicles while scattered stem cells attached to the basal membrane that separates epidermis from dermis (basal keratinocytes) are involved in replacement of interfollicular epidermis [80]. Sebaceous glands are maintained by cells at the base of each gland [81], but their niche is still largely unknown.
- While our knowledge of brown, white and beige adipose tissue is rapidly increasing, little is still known about marrow adipose tissue and its progenitors, despite recent studies demonstrating possible roles for marrow adipose tissue in regulating the hematopoietic space [82]. Inconclusive results have been published about the in situ location or “niche” of adipocyte progenitors (APs). Regardless of the high vascularity of white adipose tissue (WAT), it has also been reported that only a fraction of cells with markers of APs are found in close proximity to blood vessels [83]. Therefore, the ontogeny of WAT and the AP niche are still a matter of some debate.
- The vasculature needs to have capacity for cell turnover, growth, and repair to maintain normal homeostasis. It has emerged during the past decade that there exists an array of ancestral progenitor cells resident within the mural layers of macro- and micro-vessels [84,85]. These consist of lineage-committed endothelial progenitor cells (EPCs) [86] and smooth muscle progenitor cells (SPCs) [87], multipotent vascular stem cells (MVSCs) [88], mesenchymal stem/stromal cells (MSCs) [89], adventitial macrophage progenitor cells (AMPCs), and circulation-derived hematopoietic stem cells (HSCs) [90]. The inner adventitia, adjacent to the external elastic lamina, has emerged as the prime candidate for the vascular stem cell niche.
- In the heart, the myocardium lacks the basal-apical orientation typical of epithelial organs, making it difficult to delineate the precise localization of cardiac stem cell (CSC) niches. The epicardial lining has been employed to define anatomically several classes of niches in the adult heart [91,92,93,94,95,96]. However, cardiac niches are not limited to the subepicardium and are dispersed throughout the myocardium. CSC niches are more numerous in the atria and apex, which represent protected anatomical areas characterized by low hemodynamic stress [97,98]. Recently, these CSC have been put into controversy: a study provided in vivo genetic evidence for nonmyocyte to myocyte conversion in embryonic but not adult hearts, arguing again the myogenic potential of putative stem cell populations for cardiac regeneration in the adult stage [99].
- Regarding the central nervous system, several researchers have identified the lateral subventricular zone (SVZ) and in the subgranular zone (SGZ) of the dentate gyrus within the hippocampus [100,101,102]. Astrocytes in SVZ and SGZ are able to give rise to neuroblasts and subsequently mature neurons. However, the presence of a stem cell niche in the adult human brain is under debate [103,104]. Considering the hypoxic nature of human brain, it is conceivable that neural stem cells (NSCs) in the brain would be located in a relatively hypoxic environment. When it comes to embryonic development and early stages of life, there is evidence that cell fate decision in neural stem cells (NSCs), which can generate both neurons and glia, is affected by oxygen tension [105].
- The liver has a high regenerative capacity that involves stem/progenitor cells when the proliferation of hepatocytes is impaired. Liver stem/progenitor cells, termed hepatic progenitor cells (HPCs) [106], emerge when hepatocyte proliferation is overwhelmed by persistent or severe liver injury. There is evidence that hepatic progenitor cells can originate from niches in the canals of Hering; in addition, the space of Disse may also serve as a stem cell niche during foetal haematopoiesis and constitute a niche for stellate cells in adults [107].
- The existence, phenotype, and anatomical location of stem/progenitors in the adult pancreas are actively debated [108]. Although some reports claim the existence of multipotent stem cells within the pancreas [109], most suggest that these cells are rare in the postnatal pancreas [110]. Ongoing studies suggest that postnatal pancreatic stem cells (PSCs) reside within the biliary tree, primarily the hepato-pancreatic common duct, and are rare in the pancreas proper [111].
- In adult kidneys, it has been proved that, after an injury, tubules can recover completely, but this is not the case for nephrons, which are not able to regenerate. Several cellular types with stem cell properties have been isolated from human adult kidneys [112,113]. These cells have been identified as a subset of parietal epithelial cells (PEC) in the Bowman’s capsule, which exhibits coexpression of the stem cell markers CD24 and CD133. However, their ability to differentiate and form new tissue in vivo is less studied and still controversial.
- Turnover of the epithelial cell lineages within the gastrointestinal tract is a constant process under normal homeostasis and increases after damage. This process is regulated by multipotent stem cells, which give rise to all gastrointestinal epithelial cell lineages and can regenerate whole intestinal crypts and gastric glands. The stem cells of the gastrointestinal tract are yet undefined, although it is generally agreed that they are located within a ‘niche’ in the intestinal crypts and gastric glands [114]:
  ○ Two niches seem to co-exist in the gastric unit: one in the isthmus region and the other at the base of the gland, although the precise features of the cell populations and the two niches are currently under debate [115]. The current evidence suggests that gastric stem cells in every gastric gland give rise to four functionally distinct cell lineages: parietal, surface mucous (pit), zymogenic, and enteroendocrine.
  ○ Nearly 90% of the intestinal epithelium is replaced every 3–4 days by cells newly generated from the crypt epithelium; however, long-lived intestinal stem cells (ISCs) are harboured in the crypt bottom interdigitated between Paneth cells, where cells are physically shielded from the content of the lumen [116]. To replenish the large amount of disposable functional epithelium, ISCs produce rapidly cycling progenitor cells, referred to as transit-amplifying (TA) cells. As they proliferate, TA cells migrate up the crypt-villus axis and differentiate into mature epithelial cells that are eventually shed off into the lumen [117].
- Human endometrium is the mucosal lining of the uterus and is a highly regenerative tissue, undergoing more than 400 cycles of proliferation, differentiation, and shedding during a woman’s reproductive life. During the last 10 years, an MSC subpopulation has been identified and characterized in human endometrium and in menstrual blood. Endometrial mesenchymal stem/stromal cells (eMSCs) are easily isolated from endometrial biopsy tissue [118].
- In the muscle, stem cells, known as satellite cells, are located along muscle fibre tracts attached to the plasma membrane that surrounds each muscle fibre bundle. In this case, the basal lamina may represent the niche for satellite cells [119,120].
- In bone marrow, hematopoietic stem cells (HSCs) reside along the endosteal surface close to osteoblastic cells [121,122] and in proximity to the blood vessels [123,124]. According to Keeley and Mann, both MSCs and HSCs originate from the bone marrow, but their sites of action extend throughout the organism. Indeed, it has been postulated that changes in partial oxygen pressure as cells exit the marrow into the systemic bloodstream serve as a key trigger for terminal differentiation into one cell type or another. For example, osteogenic and adipogenic differentiation of MSCs is hampered under low oxygen pressure, whereas chondrogenesis may be enhanced [125].
- Oral tissues, including tooth, periodontal ligament, and gingiva are also an important source of MSCs. Oral MSCs involve dental pulp stem cells (DPSCs), stem cells from exfoliated deciduous teeth (SHED), periodontal ligament stem cells (PDLSCs), dental follicle stem cells (DFCs), stem cells from apical papilla (SCAP) and gingival stem cells (GMSCs) [126].

Considering that the term “niche” refers to an isolated microenvironment, it is logical to accept that low PO_2_ should be recorded inside them regardless of the tissue. Sadly, to our knowledge, the exact PO_2_ inside the human niches in vivo can not be recorded with the techniques we currently have. The closest approximations have been performed in human bone marrow aspirates, where PO_2_ levels are around 5% O_2_. Thus, it is generally accepted an average PO_2_ of 3–6% O_2_ for human stem cell niches.

From a developmental point of view, the fact that adult niches remain at low oxygen pressures correlates with the PO_2_ values recorded in embryos, where embryonic stem cells (ESCs) develop and give rise to cells of all three germ layers. The preimplantation human embryo and blastocyst develop under relatively low oxygen concentrations in vivo, approximating 2–9% O_2_ [127]. The effect of oxygen on preimplantation embryos has been comprehensively examined in several species, including the human [128]. While embryos are capable of developing under a 20% O_2_ atmosphere, studies have demonstrated compromised embryo development and viability under these conditions [129,130,131]. Thus, by residing in these anatomical compartments that experience relatively low oxygen tensions, stem cells maintain a selective advantage that is well suited to their particular biological roles [132].

## 3. Oxygen Alterations In Vitro Affects Many Stem Cell Parameters

**Figure 2:**
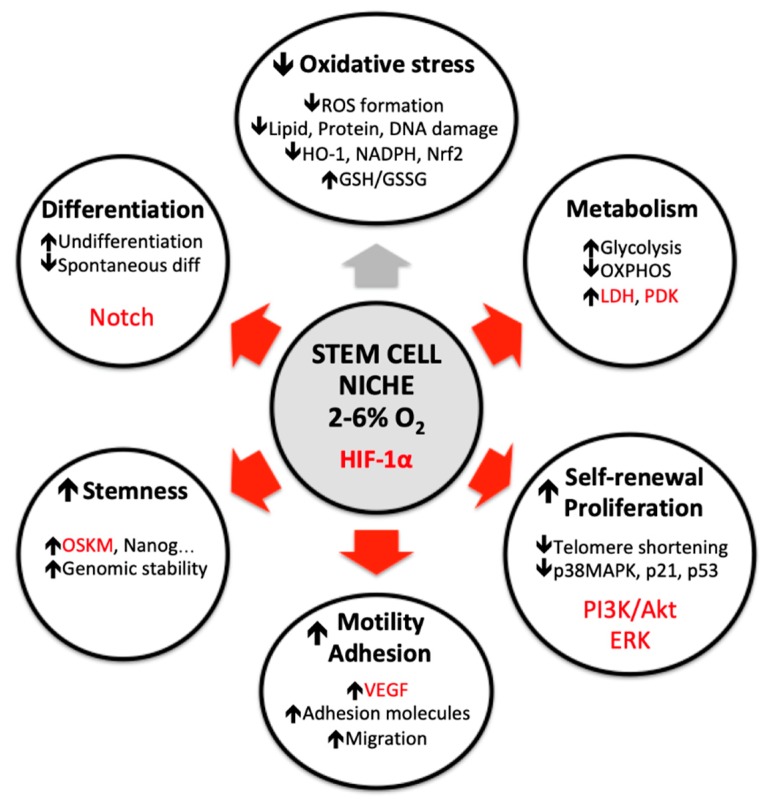
Benefits of low oxygen tension on stem cells behaviour. HIF-1α implication is shown in red. Abbreviations: HIF-1α: hypoxia inducible factor 1α; ROS: reactive oxygen species; HO-1: Heme Oxygenase 1; Nrf2: nuclear receptor factor 2; GSH/GSSG: glutathione ratio; OXPHOS: oxidative phosphorylation; LDH: lactate dehydrogenase; PDK: pyruvate dehydrogenase kinase; MAPK: mitogen activated protein kinase; PI3K: phosphoinositide 3 kinase; ERK: extracellular signal regulated kinase; VEGF: vascular endothelial growth factor; OSKM: Oct3/4, Sox2, Klf4 and c-Myc; HIF: hypoxia inducible factor.

When stem cells are cultured at an oxygen level which is not the same as the one offered by the niche microenvironment, the cells undergo a set of alterations, such as oxidative stress, metabolism turnover, reduced proliferation and self-renew, hampered motility, altered differentiation potential and a stemness potential loss. All of these consequences can be avoided if stem cells are cultured at their physiological oxygen level, as detailed below. Figure 2 summarizes the benefits that the niche microenvironment offers to their resident stem cells.

### 3.1. Reactive Oxygen Species (ROS) Formation and Antioxidant Defense

Reactive oxygen species (ROS) play an important role in determining the fate of normal stem cells because they are known to be intracellular messengers. Thus, low levels of ROS are required for stem cells to maintain quiescence and self-renewal. Otherwise, increases in ROS production can cause stem cell proliferation/differentiation, senescence and apoptosis in a dose-dependent manner, leading to their exhaustion. Therefore, the production of ROS in stem cells is tightly regulated to ensure that they have the ability to maintain tissue homeostasis and repair damaged tissues for the life span of an organism [133].

All normal stem cells appear to be highly sensitive to ROS and oxidative stress because of their relatively undifferentiated state with a long division potential for accumulating genetic damage. In fact, it has been demonstrated that high oxygen concentrations can cause oxidative stress via production of ROS that can damage lipids, proteins and DNA, and altering cell metabolism in general [134].

A study found that there was a higher formation of superoxide anion (O_2_^−^) and hydrogen peroxide (H_2_O_2_) in progenitor cells from the umbilical cord blood cultured at 20% O_2_ compared to those cultured at 5% O_2_ [135]. A more recent study revealed a significant increase in ROS formation in human dermal fibroblasts, as demonstrated by higher O_2_^−^ levels when cells where cultured at 21% in comparison with 5% O_2_ [136]. Furthermore, we recently establish an increase in ROS production (H_2_O_2_ levels detected by dihydrorhodamine-123), malondialdehyde (MDA) and carbonylation levels as well as a disruption in mitochondrial membrane potential in DPSCS cultured at 21% O_2_ compared to 3% O_2_ [137,138]. An increase in ROS production should be followed by an increase in the antioxidant defense to alleviate the oxidative stress. As expected, the analysis of glutathione redox status in these studies showed lower oxidized glutathione (GSSG) levels, higher reduced glutathione (GSH) levels and higher GSH/GSSG ratios under hypoxia (versus normoxia). Recently, we discovered an increase in HO-1 and NQO-1 protein expression in DPSCs cultured at 21% compared to 3% O_2_ which provides the evidence that the Nrf2 defense pathway is upregulated in the atmospheric oxygen condition [137].

When cells are cultured at low oxygen tension, any available oxygen diffuses to the mitochondria, creating an environment within the cytosol that lacks oxygen, thereby inhibiting the activity of prolyl hydroxylases that regulate the activation of hypoxia-inducible factors (HIFs). HIF is a heterodimer consisting of an oxygen-regulated α subunit (1α and 2α) and a constitutively expressed β subunit. The biology of the α subunits has expanded in the past years from their original role in angiogenesis to their current position in the self-renewal, stemness and differentiation of stem cells. In low oxygen conditions, HIF-1α is not hydroxylated and therefore is stabilized to initiate HIF transcriptional activity [139].

### 3.2. Metabolism

Recently, we have expanded our understanding of stem cells metabolism and how metabolic pathways may affect homeostasis and quiescence. Due to the low oxygen availability, these cells must rely heavily on anaerobic glycolysis, rather than mitochondrial oxidative phosphorylation (OXPHOS), to support ATP production [140,141,142]. However, low OXPHOS in HSCs is in part related to cell-specific mechanisms rather than only reflecting an environmental adaptation to low oxygen [143].

Moreover, self-renewing HSCs need to limit mitochondrial respiration to remain in a quiescent state [144,145,146]. When they are prone to differentiate, a rapid switch to mitochondrial OXPHOS is observed, probably to meet the robust energy demands associated with differentiation [147,148].

A key player in the regulation of stem cell metabolism is HIF1α, a transcription factor involved in the cellular responses to low oxygen availability [149]. Cells cultured at low oxygen tension express HIF1α, which activates multiple glycolytic genes, such as lactate dehydrogenase (LDH) or pyruvate dehydrogenase kinase (PDK), making stem cell metabolism more similar to the one present in their in vivo niche [150].

When stem cells that reside in a hypoxic niche are exposed to atmospheric oxygen levels, they are forced to activate a cellular response in which oxygen consumption by OXPHOS is increased and glycolysis decreased. This switch in the metabolism is detrimental for cellular function, as it promotes oxidative damage, senescence, genomic instability and decreases lifespan [151].

To summarize, stem cells generally rely on glycolysis (low oxygen), rather than their committed progeny, which is typically more oxidative (higher oxygen). Thus, monitoring oxygen levels is a critical step, especially when differentiation of the stem cell culture is required.

### 3.3. Self-Renewal and Proliferation Rate

Many studies have observed the low proliferation rate at the environmental oxygen tension compared to the physiological one in many types of stem cells: neuronal stem cells (NSCs) [152]; bone marrow stem cells (BMSCs) [153,154], umbilical cord stem cells (UCSCs) [155]; adipose-derived stem cells (ADSCs) [156,157] muscle precursor cells [158,159], and also in human fibroblasts WI-38 [160]. The last study attributed the low proliferation rate of the fibroblasts cultured at the environmental oxygen tension to the telomere shortening while another study correlated it with DNA damage [161] as chromosomal integrity has been directly related to oxidative stress [162,163]. A similar study revealed that hypoxic conditions induce an immediate and concerted downregulation of genes involved in DNA repair and damage response pathways (MLH1, RAD51, BRCA1, and Ku80), concomitantly with the occurrence of microsatellite instability while maintaining telomere length [164].

Focusing on cell cycle regulation, Lees et al. observed a 2-fold increase in p21^Waf1/Cip1^ protein and a 2.7-fold increase in its mRNA after 48 h in 20% O_2_ compared to 5% O_2_, while there was surprisingly no difference in p21^Waf1/Cip1^ promoter activity. Oxidative stress-induced p21^Waf1/Cip1^ overexpression generally acts via a p53-dependent mechanism [165,166]. It has been shown that p53 phosphorylation increases in cultures maintained at 20% O_2_ resulting in cell-cycle arrest, decreased proliferation, and differentiation of NSCs toward the glial lineage [167,168]. Nevertheless, there is also a p53-independient pathway [169] where the p38MAPK phosphorylates p21^Waf1/Cip1^ in response to oxidative stress stimuli by inducing phosphorylation at Ser130 in vitro and in vivo [170].

Many researchers have demonstrated that hypoxia or hypoxia-inducible factor 1 alpha (HIF-1α) stabilization improves several MSC functions, including cell adhesion, migration, and proliferation, thereby increasing their therapeutic potential [171]. For example, the paracrine effect of ADSCs is enhanced under hypoxic conditions, where HIF-1α is more stable and it increases secretion of vascular endothelial growth factor (VEGF), thereby improving the regenerative potential of ADSCs [172]. Furthermore, the enhancement of the proliferation capacity of human umbilical cord blood-derived MSCs by hypoxia is now known to be dependent on the expression of HIF-1α and the ERK signalling pathway [173].

The human genome encodes 1048 microRNAs (miRNAs) that regulate virtually all biological processes. Recently, several hypoxia-inducible miRNAs have been described to target transcriptional activity leading to enhanced cell proliferation, migration as well as decrease in growth arrest and apoptosis through the activation of multiple signalling pathways [174]. MiR-486 expression has been described to promote proliferation, increase angiogenic activity and reduce apoptosis of BM-MSCs through a PTEN-PI3K/AKT signalling pathway [175]. Another study found that hypoxia significantly increased the expression of MVs-released miR-210 by ADSCs, which in turn significantly promoted the proliferation, migration and invasion of human umbilical vein endothelial cells (HUVECs) [176]. A mechanistic study revealed that hypoxia activates the Notch signalling pathway, which subsequently represses the expression of miR-1 and miR-206 through canonical Hes/Hey proteins, leading to increased levels of Pax7, a key regulator of satellite cell self-renewal [177], thus, suggesting that hypoxia promotes asymmetric self-renewal divisions and inhibits asymmetric differentiation divisions without affecting the overall rate of proliferation.

### 3.4. Motility and Adhesion

Wound healing is a typical condition in which epithelial, endothelial as well as mesenchymal cells are firstly subjected to activation of their motility in order to repopulate the damaged region and then they show a strong proliferative response in order to successfully complete the wound repair process [178]. Testing the hypothesis that oxygen can impair cell proliferation, survival, and migration of MSCs, a study conducted human CSCs culture at 21%, 5% and 0.5% O_2_. Their findings suggest that physiological O_2_ (5%) levels increased migration compared with room air (21%) and hypoxia (0.5%), and treatment with MSC-conditioned media rescued CSCs migration under hypoxia to levels comparable to physiological O_2_ migration [179].

A study about BMSCs [154] and another about ADSCs [157] showed that oxygen tension affected the physiological motility of the cells. In fact, the physiological oxygen tension increased the ability of the cells to migrate and upregulated the mesenchymal gene expression of fibronectin, N-cadherin (adhesion molecules) and vimentin: a mesenchymal marker and one of the fibrotic proteins which form the intermediate filaments of the intracellular cell skeleton, particularly in the embryonic stem cells [180].

As previously mentioned, VEGF expression is upregulated by hypoxia and stimulates the motility of a range of cell types, including progenitor and stem cells. Focal adhesion kinase (FAK) is a non-receptor cytoplasmic tyrosine kinase that plays a key role in the regulation of cytoskeletal reorganization, cellular adhesion, growth, survival, and migration [181]. It has been reported that VEGF stimulates FAK tyrosine phosphorylation in endothelial cells, which is associated with new focal adhesions and increased endothelial cell migration [182,183].

### 3.5. Differentiation Fate

As stated before, low oxygen tensions keep human stem cells in a self-renewable undifferentiated state. Some researchers have demonstrated that physiological oxygen levels are beneficial for the in vitro maintenance of human ESCs [184,185], neural crest stem cells (NSC) [186] and BMSC [187] due to a decrease in the amount of spontaneous differentiation supporting self-renewal. This could be explained in part by the Notch signalling pathway, which has been evolutionarily conserved to maintain stem or progenitor cell fates in multicellular organisms [188,189]. Myogenic, haematopoietic, and neuronal precursor cell differentiation is inhibited by members of the Notch family [190,191,192,193]. Similarly, it has been shown that hypoxia directly influences Notch activity mediated directly by HIF-1α. Indeed, HIF-1α has been shown to physically associate with Notch promoting its stability, thus blocking neuronal and myogenic differentiation [194].

On the other hand, it has been proved that excessive ROS results in dysfunctional differentiation of HSC [195]. In fact, studies performing in vitro differentiation of MSC into osteoblasts, adipocytes and chondrocytes show controversial results. Several studies assessing BM-MSC proved increased rates of osteogenesis [154,196,197], adipogenesis [198,199] and chondrogenesis [200,201] at low oxygen tensions. However, other studies reveal completely opposite results, where low oxygen tension impaired the tri-lineage differentiation potential of MSC, or no significant differences were found [125,196,202,203,204]. Similar results have been observed on embryonic pancreatic cells cultured at high PO_2_, where HIF-1α expression is decreased and numerous differentiated β-cells are developed [205].

All of these controversial results could be explained in part by the % O_2_ and the duration of the exposure used in their experiments. Some of these studies induced differentiation during a short-term period (less than 72 h), while others maintained the cells up to 30 days or more at low PO_2_. The oxygen pressure average in these studies ranges from 0.1% to 5%, which means that some cells were exposed to a more anoxic environment than the others.

Oxygen and ROS also play a role in neuronal differentiation and they further impact tumour growth by influencing cell proliferation and differentiation, such as in neuroblastoma development. Therefore, manipulating oxygen and ROS production represents a useful therapeutic tool if one needs either to enhance or to modulate neurogenesis and neuronal differentiation, such as in cell replacement therapies [206].

### 3.6. Stemness Maintenance

Low oxygen tension clearly promotes the undifferentiated state in several stem cells, but the molecular mechanisms underlying these observations remained obscure until recently. A link has been demonstrated between hypoxia, HIFs and molecules that are crucial for the regulation of the differentiation of stem and/or progenitor cells, including Notch, β-catenin, OCT3/4, and c-MYC. As we have previously shown, the physiological oxygen tension upregulated the four pluripotency-related genes [207,208]: SOX2, OCT3/4, KLF4 and c-MYC (OSKM) in human DPSCs [138]. This result could be explained, at least in part, by the fact that HIF-2α regulates the expression of the transcription factor OCT3/4, essential for maintaining the stemness potential [185,209,210], as well as the expression of SOX2 and Nanog which inhibit the promoter genes of differentiation [211]. It is also known that HIF-1α and HIF-2α have been shown to have opposing effects on the activity of c-MYC and thus several implications on stem cells function. HIF-1α inhibits c-MYC activity [212,213], whereas HIF-2α has been shown to promote c-MYC-dependent proliferation in renal carcinoma cells and multiple other cell lines [214].

To further define the significance of HIF-1α in MSC function, some researchers established adult BM-derived MSCs that are able to sustain high level expression of ubiquitin-resistant HIF-1α during long-term biological processes. Using this model, they showed that the stabilization of HIF-1α proteins exerts a selective influence on colony-forming mesenchymal progenitors promoting their self-renewal and proliferation, leading to the induction of pluripotent genes and the inhibition of their terminal differentiation into osteogenic and adipogenic lineages [215].

A similar study using Wharton Jelly (WJ)-MSC proved that 5% O_2_ stimulates the expression of OCT4, NANOG, SOX2 and REX1 genes, which maintains WJ-MSC in an undifferentiated state, enabling expression of stemness-related transcription factor (SRTF) genes and protein, a hallmark of de-differentiation towards more immature phenotypes. Moreover, under this condition, cells are stimulated to grow faster with formation of numerous 3D proliferation centres, another marker of undifferentiated stem cells [216].

Another aspect of stemness is genomic stability. Recent studies have described the occurrence of chromosomal abnormalities and mitochondrial dysfunction in human stem cells, particularly after extensive passaging in vitro and/or expansion under low oxygen tensions. In the bone marrow, hematopoietic and mesenchymal stem cells form a unique niche in which the oxygen tension is low. Therefore, permanent culture under low oxygen pressure should reflect the better physiological conditions. MSC cultured at 5% O_2_ for several passages were morphologically undifferentiated, contained less mitochondria and displayed a genetic program that maintained cells undifferentiated and multipotent [217].

### 3.7. Reprogramming Efficiency

The generation of pluripotent stem cells (iPSCs) from somatic cells has opened a world of possibilities in basic and applied research. However, reprogramming is a time-consuming process and efficiency is generally low, which could be a limitation in the translation to the clinic. Oxygen concentration present in the cellular microenvironment is a key factor that has shown to affect reprogramming efficiency in several ways.

For instance, Yoshida et al. found increased efficiency of reprogramming to generate iPSCs when they introduced the four transcription factors (OSKM) into mouse embryonic fibroblasts and human somatic cells cultured under 1–5% O_2_ compared to those cultured at 21% O_2_ [218]. In addition, they were able to generate iPSCs when they transduced cells with only two of the four transcription factors (OCT3/4 and KLF4) and cultured them in 5% O_2_.

As we have stated before, the metabolism of stem cells differs from that of their progeny; this aspect is critical, as reprogramming cells to pluripotency requires a shift from oxidative to glycolytic metabolism. This shift is mediated by HIF1α and HIF2α, two factors induced by low oxygen tensions, which are both necessary to initiate the metabolic switch and for acquisition of pluripotency [219,220].

Table 1 summarizes the findings of the most relevant studies culturing human stem cells at different oxygen concentrations.

## 4. Stem Cells Defense Pathways Activated by Oxygen

Both in vivo and in vitro, stem cells rely on their capacity to adapt to stress conditions. When damage accumulates, mitotic cells from renewable tissues have two mechanisms to avoid replication. They can stop cell cycle progression and enter senescence, or trigger cell death programs such as apoptosis. It is still unclear what determines if a cell undergoes senescence or apoptosis. Although most cells are capable of both, these processes seem to be exclusive [221] yet linked to each other [222]. There is a third option, autophagy (self-eating), which can lead either to cell survival or cell death. In most circumstances, autophagy promotes cell survival by adapting cells to stress conditions; however, when apoptosis is inhibited, autophagy is reportedly conducive to cell death acting as a back up mechanism [223]. Finally, autophagic cell death is reported to avoid apoptosis as well as senescence [224], suggesting a crosstalk between these three processes.

### 4.1. Autophagy

The catabolic and self-degradative process termed autophagy consists of three different forms: microautophagy, which implies the direct uptake of soluble cytosolic substrates in the lysosomes via invagination of the lysosomal membrane; chaperone-mediated autophagy, which degrades specific proteins carrying the peptide motif KFERQ by lysosomes; and macroautophagy, involving the formation of double-membrane vesicles (autophagosomes) containing an autophagic cargo and their fusion with lysosomes. Independently of the type of autophagy, the autophagic cargo is degraded by lysosomal acidic hydrolases and cathepsins and the molecules produced are released into the cytoplasm and re-used as building blocks in different anabolic pathways [225].

Basal autophagy allows the removal of redundant or damaged and potentially toxic organelles and protein aggregates, thus representing an important system for quality control in cellular homeostasis. Therefore, autophagy is different from other types of cell death but is generally regarded as a survival mechanism that is highly conserved from yeast to mammals. In addition, it has been reported that low oxygen tension can induce autophagy, which enhances both cell death and cell survival. An early induction of autophagy by low oxygen tension may be strongly linked to the self-renewal activities of MSCs [226], which could be an effective way to sustain a healthy population of stem cells via balancing abnormal cell clearance with normal cell proliferation, contributing to maintaining self-renewal activities [227]. Supporting this finding, it has been proved that culture of BM-MSCs at low oxygen tension enhances survival and viability by inducing basal autophagy through HIF-1α and the AMPK/mTOR signalling pathway [228,229].

Furthermore, damaged mitochondria by excessive ROS can be efficiently removed in stem cells via autophagy by a process called mitophagy. Autophagy is known to decrease with age, and the failure to maintain mitochondrial quality control through mitophagy may explain the organism vulnerability and dysfunction during aging [230]. As an example, failure of autophagy in physiologically aged satellite cells or genetic impairment of autophagy in young cells causes entry to senescence by loss of proteostasis, increased mitochondrial dysfunction and oxidative stress, resulting in a decline in the function and number of satellite cells [231].

Autophagy also plays a role in the differentiation process of stem cells. In a very complete set of experiments, Pan et al. demonstrated that autophagy remains at high levels in HSCs and dermal stem cells and promotes their maintenance, but, after induced differentiation, autophagic activity is downregulated. On the contrary, they proved that autophagy in NSCs, CSCs and DSCs is upregulated during their differentiation process. Furthermore, they also established that autophagy increases the reprogramming efficiency and promotes the generation of iPSCs [232]. This evidence suggests that autophagy plays a critical role in the homeostatic control of stem cell functions.

Taken together, these results demonstrate that autophagy plays a key role in stem cell survival, proliferation, differentiation and self-renewal. However, little is still known about the relation between physiological in vitro oxygen tension and autophagy, which might be of importance for stem cell therapies.

### 4.2. Apoptosis

There are two basic apoptotic signalling pathways: the extrinsic and intrinsic apoptotic pathways. The intrinsic (or mitochondrial) apoptotic pathway is triggered by a variety of intracellular stimuli, including DNA damage, growth factor deprivation, and/or oxidative stress. This pathway relies on the formation of the apoptosome, which is composed of procaspase-9, Apaf-1 and cytochrome c. A series of Bcl-2 family members control the release of cytochrome c by regulating mitochondrial membrane permeabilization. The extrinsic pathway of apoptosis is initiated by the binding of death ligands to death receptors in the TNF receptor superfamily. This interaction is followed by the assembly of the death-inducing signalling complex (DISC), which consists of FADD and procaspase-8/10. DISC then either activates downstream effector caspases-3, 6 and 7 to directly induce cell demise or cleaves the Bcl-2 family members, thereby triggering the mitochondria-mediated intrinsic apoptotic pathway [233].

A recent study compared proliferation rates of BM-MSCs cultured at 1%, 5% and 18% O_2_. As expected, cells exposed to lower oxygen concentrations displayed greater proliferative potential and reduced apoptosis, as demonstrated by a downregulation of Bax and cleaved caspase-3 expression and an upregulation of antiapoptotic protein Bcl-2 [234]. In fact, it has been proved that caspase-3 silencing modulates the cell cycle of MSCs, promotes cell proliferation and enhances the anti-apoptotic capacity of MSCs under low oxygen conditions in vitro [235].

“Hypoxic preconditioning” has been proven to be an effective method to enhance the therapeutic action of MSCs. As such, hypoxic preconditioning increased the expression of pro-survival and pro-angiogenic factors including HIF-1a, angiopoietin-1, VEGF, erythropoietin, Bcl-2 and Bcl-xL in BM-MSCs before transplantation into infarcted hearts [236]. Furthermore, the administration of hypoxic preconditioned MSCs attenuated ischemia/reperfusion injury by inhibiting inflammatory responses associated with ROS generation. Mechanistically, p38MAPK and NF-kB signalling pathways were downregulated, whereas mitochondrial cytochrome c, Bcl-2, glutathione and IL-10 were upregulated [237].

Thus, in vitro culturing at low oxygen tension enhances the capacity of MSCs to repair infarcted myocardium, which is attributable to reduced cell death and apoptosis of implanted cells, as well as increased angiogenesis, antioxidant and anti-inflammatory effects.

### 4.3. Senescence

Cellular senescence is described as a state of permanent and irreversible cell cycle arrest in response to different stress/negative stimuli. These include telomere shortening, DNA damage, oxidative stress, oncogene activity and others [238,239]. Though senescent cells are still viable and metabolically active, they are unresponsive to mitogenic or oncogenic stimulations and lack the specific functions of their lineage [240]. The cell cycle arrest in senescence occurs mostly in the G_1_ phase, distinguishing it from G_0_-arrested quiescent cells [241].

Senescent cells have both beneficial and negative effects and functions for both tissues and the whole organism. On one hand, senescence is a potential tumour-suppressing mechanism. On the other hand, excessive accumulation of senescent cells could create a pro-inflammatory environment favourable for the onset and progression of different age-related diseases, such as cancer [242].

Although the phenotype associated with cellular senescence is highly variable and heterogeneous, senescent cells show common traits. Enlarged cell body and irregular shape, increased senescence associated-β-galactosidase (SA-β-Gal) activity, decreased proliferation capacity, high levels of the CDKIs p16 and p21, and decreased mitochondrial membrane potential are common markers of cellular senescence both in vitro and in vivo [243].

Several studies show that human MSCs cultured at atmospheric oxygen tension (21% O_2_) exhibit an increase in cell senescence markers compared to those cultured at low physiological in vivo oxygen tension [244,245,246]. For instance, human DPSCs cultured at 21% O_2_ show increased levels of p16 mRNA expression and SA-β-Gal activity compared to those cultured at 3% O_2_ at several passages [138]. In this same study, cells cultured at 21% O_2_ exhibit a great decrease in mitochondria membrane potential and higher levels of ROS in comparison with cells at 3% O_2_. Similar studies with human DPSCs show significantly higher levels of p21 protein levels under 21% O_2_ through the p38 signalling pathway [137].

In addition, several studies with human ADSCs demonstrate higher levels of SA-β-Gal activity [247,248] and increased expression levels of the tumour suppressor genes p16, p21, p53 and pRb in those cells cultured at atmospheric O_2_ concentration compared to 2–5% O_2_ [249]. Related studies with human ADSCs found increased average cell size and ROS levels at ambient oxygen pressure at passages 12–21. Interestingly, they also discovered that the alteration of senescence-associated gene expression profile was more noticeable at 20% O_2_ after several passages, while the change at 5% O_2_ was less significant.

Moreover, atmospheric oxygen culture of MSCs showed increased expression of p21, Mdm2 and E2A with increased cell size while the culture of MSCs at lower oxygen concentrations exhibit activation of HIF-1α and suppression of p21, Mdm2 and E2A expressions [245]. MSCs under atmospheric conditions also cease proliferation earlier, whereas their counter partners could be further expanded without significant loss of proliferation capacity, which was driven by the p21 pathway. Additionally, bone-marrow derived MSCs cultured at 1% O_2_ showed lower levels of SA-β-Gal, p16 expression and higher proliferation capacity compared to those cultured under hyperoxic conditions [250].

Taken together, these results suggest that culturing at physiological oxygen levels delays senescence and inhibits senescence-related genes such as p21 and p16, preventing cell cycle arrest. However, the underlying mechanisms by which oxygen modulates cellular senescence are still not clear. The inhibition of cell cycle progression that accompanies senescence seems to be driven by two main pathways: p16 and p21. Both can be induced by stress such as ROS or other negative stimuli and activated by DNA Damage Response (DDR) or p38MAPK activity. By contrast, HIF-1α is known to be able to inhibit p16 and p21, thereby preventing oxidative stress-induced senescence.

## 5. Perspectives Regarding Stem Cell Culture Oxygen Condition for Stem Cell Therapy

As stated, although most stem cells are maintained under 21% O_2_, this is unlikely the optimal condition to preserve their stemness. As shown by our group recently, partial O_2_ pressure influences the adhesion, proliferation, and osteogenic differentiation of human dental pulp stem cells on β-tricalcium phosphate scaffold [251]. Therefore, oxygen concentration can modify stem cells behaviour when used for tissue engineering constructs for bone regeneration procedures. Other authors found similar results in an ischemia model. Cells cultured under physiological oxygen concentration (physioxia) exhibited increased proliferation, migration, and angiogenesis, and decreased senescence and apoptosis. Physioxia is a more effective environment to culture stem cells for transplantation because it owes the maintenance of native stem cell properties [247].

This review has illustrated the dramatic influence that widely used environmental oxygen tension may have on the maintenance and survival of stem cells, and also provides an insight of the highly regulated network of signalling pathways that underlies the stem cells response to oxygen alterations.

We conclude that oxygen concentration is an essential factor to be considered when culturing stem cells for tissue engineering and regenerative medicine.

## Figures and Tables

**Table 1:** Summary of relevant studies on stem cells alterations at different oxygen tensions.

Cell Type	Oxygen Conditions	Duration	Affected Parameters	Ref.
C2C12 myoblasts	6% vs. 21%	72 h	ROS production, differentiation	[134]
HSCs (CD34^+^ cells)	5% vs. 21%	7 days	ROS levels, antioxidant enzymes (SOD, CAT and GPx), glutathione redox state	[135]
Human Dermal Fibroblasts (HDFs)	5% vs. 21%	72 h	ROS production, enzymatic and non-enzymatic antioxidant response system, DNA damage, extracellular matrix (ECM) proteins	[136]
DPSCs	3% vs. 21%	Up to passage 25	Oxidative stress parameters (ROS, MDA, carbonylation, antioxidant defenses), proliferation, stemness (OSKM)	[138]
MSCs from adipose tissue	3% vs. 20%	Up to 22 passages	Genetic stability, glycolytic function, cell differentiation and ROS production and targets (Protein carbonylation and MDA)	[151]
NSCs	3% vs. 21%	10 days	Survival, renewal potential and differentiation	[152]
BMSCs	2% vs. 20%	12 days	Proliferation kinetics, metabolism, differentiation potential	[153]
BMSCs	1% vs. 21%	7 days	Proliferation, migration, morphology, adhesion molecules, osteogenic differentiation	[154]
MSCs from umbilical cord	1.5%, 2.5%, 5%, 21%	70 h	Proliferation, metabolism, pH, oxygen consumption	[155]
ADSCs	1% vs. 20%	72 h	Proliferation, ROS generation, migration, OSKM	[157]
Muscle Precursor Cells (MPCs)	5%, 10%, 15%, 20%	Up to passage 2	Cell cycle regulation (p21 and p27), Proliferation	[159]
BM-MSCs and ADSCs	2% vs. 21%	Up to passage 10	Morphology, differentiation potential, genomic stability, telomere length, mitochondrial membrane potential, ATP content	[164]
Central Nervous System (CNS) Precursor Cells	2%, 5%, 20%	Up to passage 2 (35 days)	Proliferation, HIF1α, apoptosis, multilineage differentiation potential	[167,168]
MSCs from umbilical cord	3% vs. 21%	Up to passage 12	Proliferation, HIF1α, ERK signalling pathway, stemness (OCT3/4 and Nanog), p21, p16, p53	[173]
BM-MSCs	5% vs. 21%	Up to passage 15	Donor age, differentiation potential, SA-β-Gal, miRNA sequencing, KEGG signalling pathways	[174]
BM-MSCs	1% vs. 21%	Up to passage 4	Migration, proliferation, apoptosis, differentiation potential, PTEN-PI3K/AKT signalling pathway, miRNAs, HGF and VEGF	[175]
Satellite Cells	1% vs. 21%	48 h	Quiescence, self-renewal, miRNAs, Notch signalling pathway, transplantation efficiency	[177]
CSCs	0.5%, 5%, 21%	Up to passage 10	Proliferation, survival, migration, SA-β-Gal, apoptosis	[179]
MSCs from umbilical cord	2.2% vs. 21%	24 h	ROS levels, migration, HIF1α, VEGF	[182]
ESCs	1–5% vs. 21%	Up to passage 50	Morphology, colony growth, differentiation, hGC production, embryoid body formation	[184]
ESCs	4% vs. 20%	Up to passage 50	Morphological differentiation, microarray and transcriptome profiling, HIF, stemness	[185]
Neural Crest Stem Cells	5% vs. 20%	12 days	Survival, proliferation, multilineage differentiation	[186]
BM-MSCs	1, 3, 5, 10% vs. 21%	7 days	Viability, proliferation, self-renewal, osteogenic differentiation	[187]
C2C12 myoblasts, Satellite Cells and NSCs	1% vs. 21%	7 days	Notch signalling pathway, undifferentiated state maintenance	[194]
BM-MSCs and HSCs	5, 12, 20%	10 days	ROS content, proliferation, directional differentiation, apoptosis, cell cycle, migration	[195]
BM-MSCs	2% vs. 18%	2 weeks	Osteogenic and adipogenic differentiation, HIF1α, VEGF	[196]
BM-MSCs	1% vs. 21%	7 days/4 weeks	Proliferation, migration, stemness (OCT3/4, Nanog, SALL4, KLF4), differentiation	[154]
MSCs	2% vs. 20%	7 days	Proliferation, osteogenic differentiation	[197]
BM-MSCs	0.2% vs. 21%	7 or 14 days	Osteogenic and adipogenic differentiation, HIF1α	[198]
MSCs	1, 2, 3, 4, 6% vs. 21%	2, 4, 8, 24, 48, 72 h	Adipogenic differentiation	[199]
BM-MSCs	3% vs. 21%	Isolation and expansion (4 weeks)	Chondrogenic differentiation, cell surface markers, ECM formation, expansion, HIFs	[200]
BM-MSCs	2% vs. 20%	14 days	Chondrogenic differentiation	[201]
MSCs	1% vs. 21%	21 days	Osteogenic differentiation, HIFs	[202]
WJ-MSCs	3% vs. 21%	Up to passage 13	Growth kinetics, SA-β-Gal, differentiation, HIFs, p16, p21, p53, karyotype	[203]
ADSCs	1% vs. 21%	Up to passage 2	Proliferation, multilineage differentiation, stemness (Nanog, SOX2)	[204]
ESCs (dorsal pancreatic bud)	3%, 8%, 21%	24h or 7 days	Cell differentiation, HIF1α gene and protein expression	[205]
ESCs	3–5% vs. 20%	Up to passage 3	Morphology, proliferation, pluripotency (SOX2, Nanog and OCT3/4), HIFs	[210]
BM-MSCs	1% vs. 21%	14 days	Proliferation, differentiation, self-renewal	[215]
WJ-MSCs	5% vs. 21%	2-4 weeks	Proliferation, stemness (OCT3/4, Nanog, REX1 and SOX2), HIFs, differentiation	[216]
BM-MSCs	5% vs. 21%	Up to passage 2	Morphology, differentiation, transcriptional profiling, metabolism, adhesion	[217]
Dermal Fibroblasts into IPSCs	1%, 5%, 21%	40 days	Efficiency of reprogramming into iPSCs (ESC markers, teratoma formation)	[218]
Fibroblasts, ESCs and IPSCs	2%, 5%, 21%	2 weeks	Reprogramming efficiency, HIFs, metabolism (OCR and ECAR)	[219,220]

Abbreviations: HSC: haematopoietic stem cell; HDF: human dermal fibroblast; DPSC: dental pulp stem cell; MSC: mesenchymal stem cell; NSC: neural stem cell; BMSC: bone marrow stem cell; ADSC: adipose derived stem cell; MPC: muscle precursor cell; BM-MSC: bone marrow mesenchymal stem cell; CNS: central nervous system; CSC: cardiac stem cell; ESC: embryonic stem cell; WJ-MSC: Wharton Jelly mesenchymal stem cell; iPSC: induced pluripotent stem cell.
